# Supplementary material for: 1-Nitro-2-Phenylethane as a Multitarget Candidate for Cognitive and Psychiatric Disorders: Insights from In Silico and Behavioral Approaches
Source: Pharmaceuticals (Basel). 2025 Oct 9;18(10):1511. doi: 10.3390/ph18101511 (PMC12567097; doi:10.3390/ph18101511)
Supplement: Supplementary file 1 [file pharmaceuticals-18-01511-s001.zip › pharmaceuticals-3906775-supplementary.pdf]

A   B

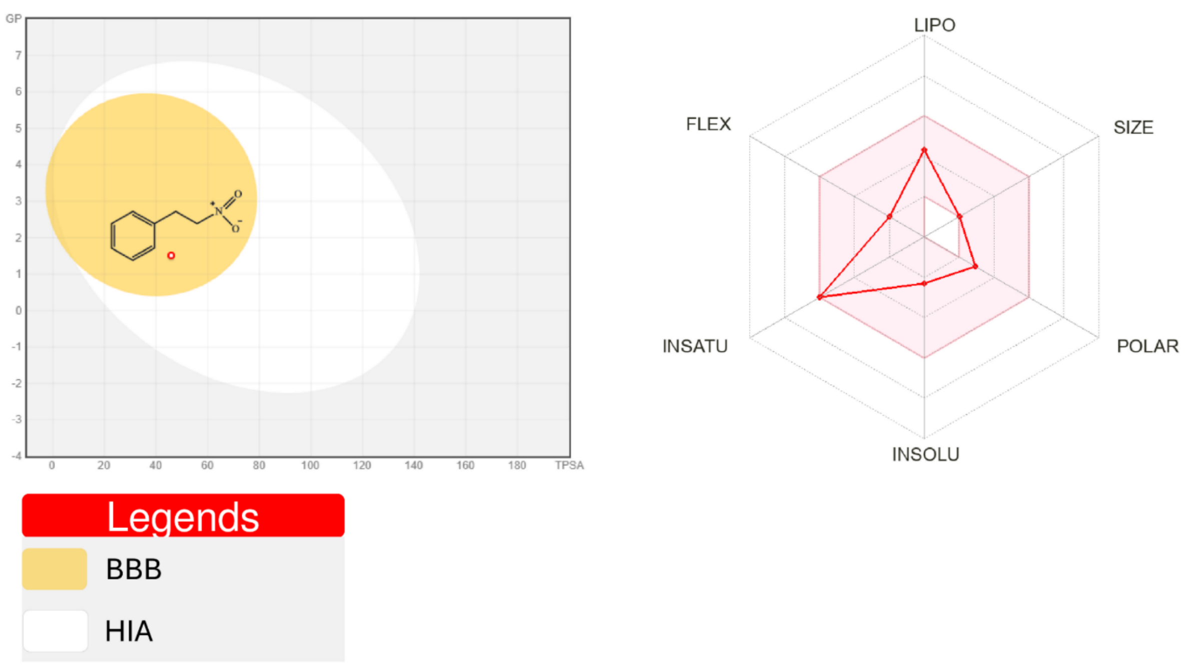

Figure S1. BOILED-Egg and Bioavailability Radar representation of 1N2PE. A represents the BOILED-Egg visualization, indicating that 1N2PE has high intestinal absorption and can easily cross the blood-brain barrier. **B** illustrates the Bioavailability Radar, showing that 1N2PE has adequate physicochemical properties, conferring drug-likeness characteristics to this drug candidate.

Table S1. Raw data from behavioral tasks.

LATENCY TO FIND THE PLATFORM IN THE TRAINING SESSIONS

TRAIL 1

CONTROL	SCOPOLAMINE	SCOPOLAMINE+1N2PE
17	60	60
60	25	21
57	57	60
22	40	60
60	60	41
58	60	60
14	24	44
33	37	60
**TRAIL 2**<br>**CONTROL**	**SCOPOLAMINE**	**SCOPOLAMINE+1N2PE**
2	43	43
38	27	22
18	23	29
4	60	51
3	17	53
12	60	16
20	60	52
7	25	9
**TRAIL 3**<br>**CONTROL**	**SCOPOLAMINE**	**SCOPOLAMINE+1N2PE**
23.5	60	30.86
29.8	60	60
49.18	60	10.87
28.01	60	60
28.19	60	12.29
19.2	60	5.51
60	60	8.64
60	60	18.95
**TRAIL 4**<br>**CONTROL**	**SCOPOLAMINE**	**SCOPOLAMINE+1N2PE**
53.55	46.47	23.8
60	49.35	60
60	49.92	15.8
7.04	27.53	60
57.09	41.44	19.2
13.98	38.73	4.36
10.49	60	38.69
11.22	60	10.49
**DISTANCE TO FIRST ENTRY TEST**<br>**CONTROL**	**SCOPOLAMINE**	**SCOPOLAMINE+1N2PE**
0.25	3.198	2.49
1.42	1.765	0.8
1.45	3.751	0.69
1.11	5.213	0.6

1.4<br>1.29<br>0.81<br>1.37	10.891<br>13.034<br>1.237<br>2.006	0.22<br>0.68<br>0.83<br>0.59
**MEAN SPEED TEST CONTROL**	**SCOPOLAMINE**	**SCOPOLAMINE+1N2PE**
0.233<br>0.23<br>0.225<br>0.218<br>0.207<br>0.229<br>0.208<br>0.217	0.298<br>0.288<br>0.26<br>0.257<br>0.272<br>0.292<br>0.257<br>0.299	0.344<br>0.338<br>0.434<br>0.353<br>0.304<br>0.422<br>0.403<br>0.323
**MAXIMUM SPEED TEST CONTROL**	**SCOPOLAMINE**	**SCOPOLAMINE+1N2PE**
0.47<br>0.411<br>0.407<br>0.391<br>0.48<br>0.431<br>0.373<br>0.426	0.413<br>0.522<br>0.461<br>0.415<br>0.448<br>0.449<br>0.459<br>0.422	0.694<br>0.567<br>0.602<br>0.621<br>0.65<br>0.594<br>0.738<br>0.67
**%IMMOBILITY TIME TRAIL 1 CONTROL**	**SCOPOLAMINE**	**SCOPOLAMINE+1N2PE**
1.666666667<br>0<br>0<br>1.666666667<br>0<br>0<br>0<br>0	0<br>0<br>0<br>0<br>0<br>0<br>0<br>0	0<br>0<br>0<br>0<br>0<br>0<br>0<br>0
**TRAIL 2 CONTROL**	**SCOPOLAMINE**	**SCOPOLAMINE+1N2PE**
0<br>0<br>0<br>0<br>0<br>5<br>1.666667<br>5	0<br>0<br>0<br>0<br>0<br>0<br>0<br>0	0<br>0<br>0<br>0<br>0<br>0<br>0<br>0
**TRAIL 3 CONTROL**	**SCOPOLAMINE**	**SCOPOLAMINE+1N2PE**
11.2766<br>5.153335<br>10.2349<br>8.845059	5.317974<br>9.173479<br>10.96602<br>13.16932	9.57046<br>5.808477<br>6.899724<br>5.985584

9.706546<br>10.85327<br>10.03902<br>5.734485	4.227346<br>7.249961<br>4.746785<br>11.26906	8.950366<br>6.25<br>8.130455<br>8.126649
**TRAIL 4<br>CONTROL**	**SCOPOLAMINE**	**SCOPOLAMINE+1N2PE**
6.255836<br>6.440044<br>5.823262<br>8.337712<br>7.120743<br>3.433476<br>9.914204<br>6.585708	7.784431<br>9.314904<br>8.60879<br>14.29971<br>8.597986<br>9.008189<br>10.9917<br>10.51454	2.731092<br>11.06862<br>6.963714<br>7.848101<br>12.31884<br>7.916667<br>9.097958<br>6.959009
**TEST<br>CONTROL**	**SCOPOLAMINE**	**SCOPOLAMINE+1N2PE**
0<br>0<br>0<br>0<br>0<br>0<br>0<br>0	0<br>0<br>3.333<br>1.66666<br>3.3333<br>0<br>1.666666<br>1.66666	0<br>0<br>0<br>0<br>0<br>0<br>0<br>0
**MEAN DISTANCE TO THE BORDER TRAIL 1 CONTROL**	**SCOPOLAMINE**	**SCOPOLAMINE+1N2PE**
0.124<br>0.088<br>0.081<br>0.098<br>0.112<br>0.091<br>0.093<br>0.119	0.111<br>0.115<br>0.127<br>0.11<br>0.13<br>0.133<br>0.115<br>0.121	0.112<br>0.114<br>0.113<br>0.109<br>0.116<br>0.118<br>0.113<br>0.113
**MEAN DISTANCE TO THE BORDER TRAIL 2 CONTROL**	**SCOPOLAMINE**	**SCOPOLAMINE+1N2PE**
0.089<br>0.089<br>0.086<br>0.196<br>0.192<br>0.109<br>0.136<br>0.123	0.088<br>0.106<br>0.036<br>0.027<br>0.073<br>0.06<br>0.095<br>0.069	0.132<br>0.122<br>0.12<br>0.12<br>0.123<br>0.132<br>0.098<br>0.101

MEAN DISTANCE TO THE BORDER TRAIL 3 CONTROL	SCOPOLAMINE	SCOPOLAMINE+1N2PE
0.087	0.1	0.104
0.104	0.114	0.107
0.113	0.099	0.102
0.104	0.121	0.108
0.105	0.115	0.112
0.103	0.097	0.093
0.096	0.09	0.113
0.109	0.082	0.091

MEAN DISTANCE TO THE BORDER TRAIL 4 CONTROL	SCOPOLAMINE	SCOPOLAMINE+1N2PE
0.112	0.112	0.125
0.094	0.082	0.134
0.086	0.092	0.106
0.091	0.118	0.102
0.108	0.11	0.103
0.108	0.119	0.123
0.107	0.116	0.097
0.122	0.11	0.1

MEAN DISTANCE TO THE BORDER TEST CONTROL	SCOPOLAMINE	SCOPOLAMINE+1N2PE
0.08	0.081	0.12
0.086	0.077	0.119
0.091	0.082	0.115
0.098	0.076	0.113
0.099	0.081	0.126
0.079	0.08	0.107
0.086	0.075	0.113
0.083	0.079	0.116

MAXIMUM DISTANCE TO THE BORDER TRAIL 1 CONTROL	SCOPOLAMINE	SCOPOLAMINE+1N2PE
0.2	0.229	0.2
0.196	0.23	0.2
0.199	0.233	0.198
0.201	0.235	0.215
0.19	0.23	0.216
0.188	0.223	0.216
0.196	0.237	0.216
0.195	0.226	0.221

MAXIMUM DISTANCE TO THE BORDER TRAIL 2 CONTROL	SCOPOLAMINE	SCOPOLAMINE+1N2PE
0.208	0.198	0.226
0.211	0.198	0.228
0.174	0.066	0.226
0.2	0.027	0.226
0.203	0.191	0.21
0.192	0.097	0.201

0.203	0.194	0.224
0.198	0.197	0.225
**MAXIMUM DISTANCE TO THE BORDER TRAIL 3 CONTROL**	**SCOPOLAMINE**	**SCOPOLAMINE+1N2PE**
0.2	0.214	0.2
0.2	0.199	0.201
0.2	0.2	0.199
0.2	0.223	0.204
0.2	0.199	0.199
0.204	0.199	0.201
0.197	0.209	0.224
0.199	0.168	0.251
**MAXIMUM DISTANCE TO THE BORDER TRAIL 4 CONTROL**	**SCOPOLAMINE**	**SCOPOLAMINE+1N2PE**
0.205	0.197	0.246
0.204	0.207	0.245
0.207	0.206	0.211
0.206	0.206	0.236
0.201	0.205	0.235
0.201	0.179	0.241
0.2	0.2	0.201
0.2	0.206	0.242
**MAXIMUM DISTANCE TO THE BORDER TEST CONTROL**	**SCOPOLAMINE**	**SCOPOLAMINE+1N2PE**
0.164	0.154	0.21
0.164	0.184	0.248
0.155	0.174	0.221
0.16	0.156	0.212
0.137	0.155	0.201
0.181	0.165	0.218
0.168	0.15	0.22
0.159	0.154	0.2

Table S2. Topology archiver for molecular dynamics and free-energy calculations.

Folder / System	Files	Description / Usage
**DAT**	complex_box.top	Full topology (receptor + ligand + solvent/ions).
	ligand.top	Topology of the ligand only.
	receptor.top	Topology of the receptor only.
**GABAA**	COMPLEX.inpcrd	Initial coordinates of the system.
	COMPLEX.parm7	Topology of the complex (with solvent/ions).
	complex.prmtop	Topology of the complex (without solvent, for MMPBSA).
	ligand.prmtop	Topology of the ligand only.
	receptor.prmtop	Topology of the receptor only.
**PgHS**	COMPLEX.inpcrd	Initial coordinates of the system.
	COMPLEX_SOLV.parm7	Full topology with solvent/ions.
	complex.prmtop	Topology of the complex without solvent (for MMPBSA).
	ligand.prmtop	Topology of the ligand only.
	receptor.prmtop	Topology of the receptor only.
**SERT**	COMPLEX.inpcrd	Initial coordinates of the system.
	COMPLEX_SOLV.parm7	Full topology with solvent/ions.
	complex.prmtop	Topology of the complex without solvent (for MMPBSA).
	ligand.prmtop	Topology of the ligand only.
	receptor.prmtop	Topology of the receptor only.
**MAKE_MD**	mdin_min1 → mdin_min4	Sequence of minimizations (from strong restraints → no restraints).
	heat.in	Gradual heating (0 → 300 K) under NVT.
	density.in	Density equilibration (NPT, box/volume adjustment).
	equil.in	Final equilibration under constant T and P.
	md.in	Production run (trajectory used for analyses).
